# Design and implementation of a jellyfish otolith-inspired MEMS vector hydrophone for low-frequency detection

**DOI:** 10.1038/s41378-020-00227-w

**Published:** 2021-01-01

**Authors:** Renxin Wang, Wei Shen, Wenjun Zhang, Jinlong Song, Nansong Li, Mengran Liu, Guojun Zhang, Chenyang Xue, Wendong Zhang

**Affiliations:** 1 State Key Laboratory of Dynamic Testing Technology, North University of China, Taiyuan, China; 2 College of Underwater Acoustic Engineering, Harbin Engineering University, Harbin, China; 3 Hubei Key Laboratory of Modern Manufacturing Quantity Engineering, School of Mechanical Engineering, Hubei University of Technology, Wuhan, Hubei China

**Keywords:** Engineering, Physics

## Abstract

Detecting low-frequency underwater acoustic signals can be a challenge for marine applications. Inspired by the notably strong response of the auditory organs of pectis jellyfish to ultralow frequencies, a kind of otolith-inspired vector hydrophone (OVH) is developed, enabled by hollow buoyant spheres atop cilia. Full parametric analysis is performed to optimize the cilium structure in order to balance the resonance frequency and sensitivity. After the structural parameters of the OVH are determined, the stress distributions of various vector hydrophones are simulated and analyzed. The shock resistance of the OVH is also investigated. Finally, the OVH is fabricated and calibrated. The receiving sensitivity of the OVH is measured to be as high as −202.1 dB@100 Hz (0 dB@1 V/μPa), and the average equivalent pressure sensitivity over the frequency range of interest of the OVH reaches −173.8 dB when the frequency ranges from 20 to 200 Hz. The 3 dB polar width of the directivity pattern for the OVH is measured as 87°. Moreover, the OVH is demonstrated to operate under 10 MPa hydrostatic pressure. These results show that the OVH is promising in low-frequency underwater acoustic detection.

## Detecting submarines via biomimicry

Jellyfish sensory organs offer the inspiration for a new sensor design that can sensitively detect low-frequency sounds underwater. These capabilities are particularly for the long-range detection of submarines, which produce faint sound signatures in the 5–200 Hertz range. Researchers have struggled to develop compact ‘hydrophone’ devices that can sensitively capture such frequencies underwater, but Wendong Zhang of the North University of China and colleagues have identified a promising solution. Modeled on the pectis jellyfish auditory organ, this system features an otolith-like structure mounted on a cross-beam that produces a piezoresisitive signal in response to vibrations. Based on this concept, the researchers fabricated a device that effectively detects very faint sounds in the 20–200 Hertz range at depths of at least 1000 meters, highlighting the potential of such ‘otolith-inspired MEMS vector hydrophone’ devices.

## Introduction

Recently, long-distance and weak noise detection for submarines has become a research hot spot, which places high requirements on the low-frequency performance of hydrophones. The submarine acoustic energy of the most powerful discrete components is located in the frequency band of 5–200 Hz^1^. Vector hydrophones have become a top choice to monitor sound pressure and velocity at low frequency^2^. Many studies have focused on the study of vector hydrophones. Yildiz et al.^3^ developed hydrophone arrays of vector sensors with spacing much less than the wavelength. Ma et al.^4^ reported a two-axis slim fiber laser vector hydrophone with a V-shaped flexed beam as the mass-spring element. Di Iorio et al.^5^ developed hydrophones to detect cracking sounds through nonintrusive monitoring of bivalve movement. Heerford et al.^6^ introduced a novel fiber hydrophone without any electrical power that can be applied to a hydrophone towed array. The volume of those vector hydrophones is usually large. On the other hand, hydrophones with miniaturized volumes and high sensitivity have become a trend^7^. Lee et al.^8^ presented a kind of MEMS piezoelectric flexural-mode hydrophone with air backing. They also developed a micromachined hydrophone employing a piezoelectric body on the gate of a field-effect transistor^9^. Xu et al.^10^ presented a kind of AlN-on-SOI micromachined hydrophone with high sensitivity. However, these piezoelectric hydrophones were nondirectional and could bear only low hydrostatic pressure due to the sealed membrane structure. This means that the work depth was limited to a low range, which was reported as 100 m^9^. Ganji et al.^11^ designed a MEMS piezoelectric vector hydrophone based on a cilium structure. Amiri et al.^12^ designed a flat cap mushroom-shaped MEMS piezoelectric hydrophone. These kinds of hydrophones were directional and could bear relatively high hydrostatic pressure due to the open structure. However, they have not been fabricated and measured so far, and only the design and simulation have been completed. Generally speaking, MEMS piezoelectric hydrophones are considered more sensitive than other hydrophones, whether nondirectional or directional, measured or in design. The main concern is that the fabricated devices are based on sealed membrane structures, bringing high sensitivity but low work depth. MEMS piezoresistive vector hydrophones have been developed that have the advantages of low-frequency operation and miniaturized detection^13^. They were also proven to be able to resist high hydrophone pressure due to their open structure. Much work has been done in this area, including microstructure parameter optimization, cilium optimization, and package optimization. A whisker-inspired MEMS vector hydrophone (WIVH) was proposed, which was encapsulated with Parylene, in order to improve the sensitivity–frequency response performance^14^. A cup-shaped MEMS vector hydrophone (CuVH) was presented with an improved sensitivity of −209.2 dB@100 Hz (0 dB@1 V/μPa)^15^. A lollipop-shaped MEMS vector hydrophone (LVH) was developed, and its sensitivity reached −205 dB@100 Hz (0 dB@1 V/μPa)^16^. How can the performance of vector hydrophones be further improved in the low-frequency range?

**Fig. 1:**
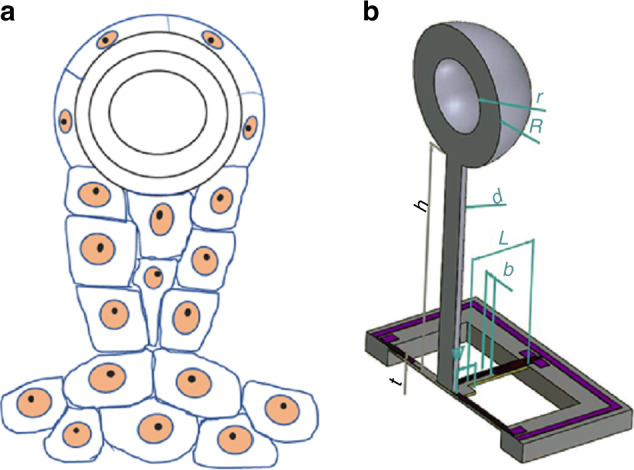
Microstructure models of auditory organs of pectis jellyfishes and OVH. **a** Auditory organs of pectis jellyfishes, **b** OVH with an otolith-shaped cilium and cross beam; *b* is the width of the beam, *t* is the thickness of the beam, *l* is the length of the beam, *w* is the half-width of the mass square, *h* is the height of the rod, *d* is the radius of the rod, *R* is the outer radius of the sphere, and *r* is the inner radius of the sphere.

Auditory organs of pectis jellyfish consist of otoliths, auditory hairs, and supporting cells, as shown in Fig. 1a. The auditory organ is naturally buoyant and can respond notably strongly to the sound of ultralow frequency. Inspired by the auditory organs of pectis jellyfish, we propose a kind of otolith-inspired MEMS vector hydrophone (OVH) relying on a hollow buoyant sphere on top of the cilium, as shown in Fig. 1b. The bionic microstructure consists of two important parts: a cross-beam structure and a cilium with otolith-shaped microstructure. The cilium is mounted on the center of the cross-beam microstructure, and piezoresistors are located at the root of the beams. When an acoustic wave is applied on the cilium, the cilium vibrates, causing deformation of the cross beam. Finally, the resistances of the piezoresistors change, which is converted to voltage signals via a Wheatstone bridge. Therefore, underwater acoustic signals can be detected.

## Materials and methods

When designing the microstructure, two key problems must be taken into account: increasing the sensitivity and broadening frequency of the band. To address this issue, we must analyze the influence of the microstructure on the sensitivity and frequency bandwidth. In the acoustic-electric transduction process, the cilium plays an important role in the perception of sound waves, which transmit the vibration of medium particles to the microstructure. It is observable that the cilium structure parameters have a great influence on the performance of the hydrophone. A full parametric analysis is performed to optimize the cilium structure. In addition, the hydrophone encounters shock in launching and working procedures. It is also challenging to keep the structure robust to improve the sensitivity. Shock resistance is researched. A sketch of the microstructure dimension is shown in Fig. 1b.

### Influence on the resonance frequency

The resonance frequency is determined by the energy method. According to the law of conservation of energy, the strain energy *T*_max_ and kinetic energy *V*_max_ are constant in the case of free vibration without damping:

1 $$T_{{\mathrm{max}}} = V_{{\mathrm{max}}}$$

**Fig. 2:**
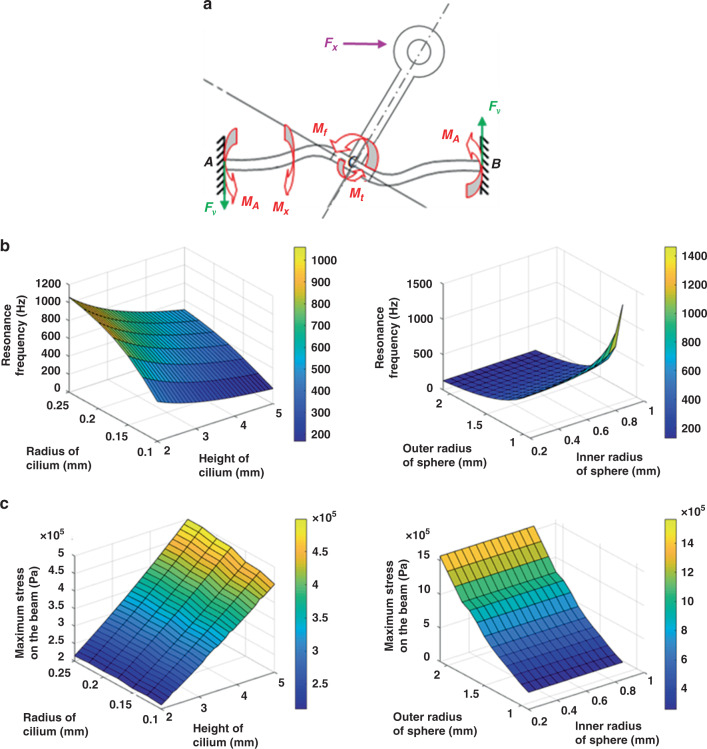
Analysis of the influence of the structure parameters on the resonance frequency and stress. **a** Mechanical analysis of the structure; **b** relationship of the resonance frequency and various structure parameters; **c** relationship of the maximum stress on the beam and various structure parameters.

When the cilium is subjected to a horizontal force *F*_*x*_, two kinds of moments arise: a flexural moment *M*_*x*_ produced by the *X*-axis beam and a torque moment *M*_*t*_ produced by the *Y*-axis beam, which remain balanced with an external force *F*_*x*_, as shown in Fig. 2a.

It can be assumed that the strain energy of the cilium and kinetic energy of the beam can be neglected.

The strain energy of the beam consists of the bending energy and torsion energy:

2 $$V_{{\mathrm{max}}} = 2 \times \left( {\frac{1}{2}{\int}_0^l {\frac{{M_x^2}}{EI}{\text{d}}x + \frac{1}{2}M_t\theta _l} } \right) = \frac{{F_v^2l^3 + 3F_vM_Al^2 + 3M_A^2l}}{{3{EI}}} + \frac{{G\beta bt^3\theta _l^2}}{l}$$

where *E* is the elastic modulus of the cantilever beam, *I* is the moment of inertia, *θ*_*l*_ is the bent angle of the mass square, *G* is the shear modulus, and *β* is the torsion coefficient.

The kinetic energy of the cilium is as follows:

3 $$T_{{\mathrm{max}}} = \frac{1}{2}\omega ^2\left[ {{\int}_0^h {\rho \pi d^2\left( {y\theta _l} \right)^2{\mathrm{d}}y + \rho \times \frac{4}{3}\pi \left( {R^3 - r^3} \right)\left( {h + R} \right)^2\theta _l^2} } \right]$$

The relationship of the resonance frequency and various structure parameters is demonstrated in Fig. 2b, which is obtained by modal analysis.

### Influence on the sensitivity

Assuming that an external pressure *P*_*x*_ is applied on the cilium along the *X*-direction, the beam is bent and compressed. The stress distribution on the cantilever beam is:

4 $$\sigma _x\left( x \right) = \frac{{3\left( {1 + v} \right)\left[ { - \left( {3l + 6w} \right)x + \left( {l^2 + 3wl} \right)} \right]}}{{2bt^2\left[ {\left( {1 + v} \right)\left( {l^2 + 3wl + 3w^2} \right) + 6\beta \left( {5l^2 + 12wl} \right)} \right]}}\left[ {dh^2 + \pi R^2\left( {h + R} \right)} \right]P_x + \frac{{\left( {2dh + \pi R^2} \right)P_x}}{{bt}}$$

where *v* is the Poisson ratio of the beam and *x* is the distance to the beam root.

The piezoresistors are distributed on the root of the beams, which are nearly the location of maximum stress. The output voltage of the Wheatstone bridge is approximately proportional to the maximum stress on the beam:

5 $$V_{o\_x} = \left\{ {\frac{{3\left( {1 + \nu } \right)\left( {l^2 + 3wl} \right)}}{{2bt^2\left[ {\left( {1 + \nu } \right)\left( {l^2 + 3wl + 3w^2} \right) + 6\beta \left( {5l^2 + 12wl} \right)} \right]}}\left[ {dh^2 + \pi R^2(h + R)} \right] + \frac{{\left( {2dh + \pi R^2} \right)}}{{bt}}} \right\}P_x\pi _lV_{{\mathrm{in}}}$$

where *π*_*l*_ is the piezoresistance coefficient and *V*_in_ is the input voltage. It can be concluded that the stress distribution is related to the size parameters of the microstructure.

Pressure loads are applied along the *Y*-direction, and the stress distribution curves with different parameters can be obtained by static analysis. The relationship of the maximum stress on the beam and various structure parameters is shown in Fig. 2c.

**Fig. 3:**
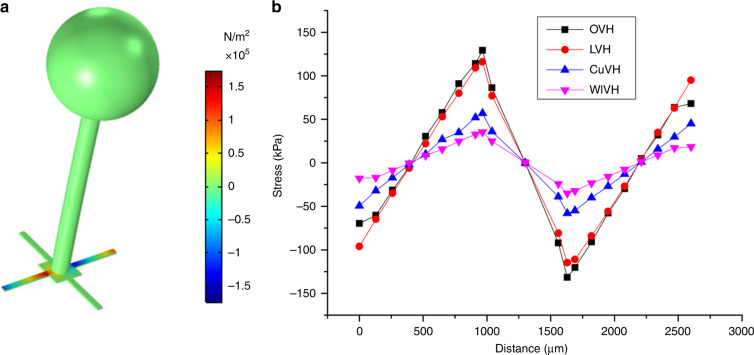
Simulation and comparison of the stress on the microstructures. **a** Stress nephogram on the cross beam of the OVH when external pressure is applied on the cilium along the *X*-direction. **b** Stress distributions on the beams of various structures. Values of the *X*-axis indicate the distance of the site on the beam from the starting point.

Taking the resonance frequency and maximum stress into consideration, the dimensions of the microstructure are illustrated in Table 1. The resonance frequency of the microstructure is 527 Hz in air and 314 Hz in water, resulting in a working bandwidth as high as 200 Hz (an analysis is shown in the Supplementary File). Moreover, the stress is optimized to realize high sensitivity. The stress nephogram on the microstructure is illustrated in Fig. 3a. The stress distributions can be obtained from the nephogram. Compared to the case of the previously presented LVH, CuVH, and WIVH, the maximum stress of the OVH is obviously higher, as shown in Fig. 3b.

**Table 1:** Dimensions of the microstructure.

Dimension	Value	Dimension	Value
Outer radius of the sphere (μm)	1000	Inner radius of the sphere (μm)	530
Radius of the rod (μm)	175	Height of the rod (μm)	3500
Thickness of the beam (μm)	40	Length of the beam (μm)	1000
Width of the beam (μm)	120	Width of the mass square (μm)	600

### Influence on the shock resistance

When the hydrophone encounters shock with acceleration *a*, the beam is bent and compressed due to the inertia force of the cilium *f*_*c*_ (the inertia force of the beam can be neglected). The maximum stress of the structure appears at the root of the silicon beam and does not exceed the fracture strength of Si (175 MPa).

When OVH encounters *X*-direction shock with acceleration *a*_*x*_, the stress analysis is similar to that of the sensitivity. In view of the shape of the cilium, the moment of inertia force is divided into two parts: the sphere and the rod, due to the different lengths of the force arm:

6 $$\sigma _{{\mathrm{x}}\_{\mathrm{max}}} = \frac{{3\left( {1 + \nu } \right)\left( {l^2 + 3wl} \right)}}{{2bt^2\left[ {\left( {1 + \nu } \right)\left( {l^2 + 3wl + 3w^2} \right) + 6\beta \left( {5l^2 + 12wl} \right)} \right]}}\left[ {\frac{1}{2}d^2h^2 + \frac{4}{3}\left( {R^3 - r^3} \right)\left( {h + R} \right)} \right]\pi \rho a_x + \frac{{\left[ {d^2h + \frac{4}{3}\left( {R^3 - r^3} \right)} \right]\pi \rho a_x}}{{bt}}$$

When the OVH encounters a *Z*-direction shock with acceleration *a*_*z*_, the inertia force of cilium is vertically applied on the central square, resulting in beam bending:

7 $$\sigma _{{\mathrm{z}}\_{\mathrm{max}}} = \frac{{3\left( {2l^2 + 2bl + bw} \right)}}{{4bt^2\left( {2l + b} \right)}}\left[ {d^2h + \frac{4}{3}\left( {R^3 - r^3} \right)} \right]\pi \rho a_z$$

**Fig. 4:**
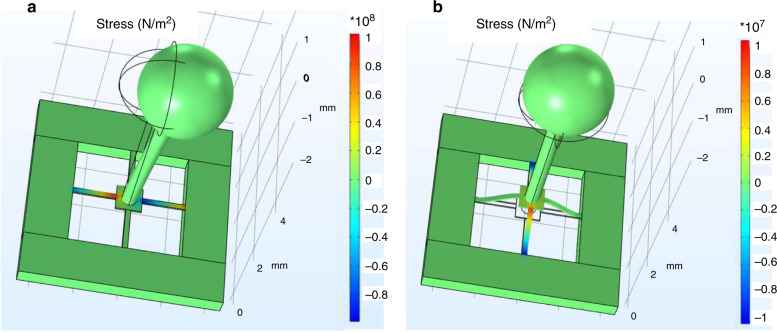
Stress nephogram under 60 g shock along diverse directions. **a** Along the *X*-direction; **b** along the *Z*-direction. The maximum stress on the beam could be extracted as 115.8 MPa along the *X*-direction and 12.5.

Stress nephograms were simulated via Comsol Multiphysics (Fig. 4), in order to find the maximum stresses of the OVH structures at an acceleration of 60 g along the *X*- and *Z*-directions, which were 115.8 and 12.5 MPa, respectively. In contrast, the corresponding maximum stresses of the LVH were 181.4 and 34.7 MPa. The corresponding maximum stresses of the CuVH were 151.7 and 18.6 MPa. The maximum stresses of the WIVH along the *X*- and *Z*-directions were 38.7 and 4.2 MPa, respectively. In contrast with the structure with the stereo cilium such as the OVH, LVH, and CuVH, the maximum stress of the WIVH was lower. It should be noted that the maximum stress of the WIVH was lower than that of the OVH by 9.5 dB, but the average equivalent pressure sensitivity of the WIVH was lower by 13.6 dB, as shown in Table 2. In addition, the OVH could resist a higher shock than the LVH and CuVH. It should be noted that the sensitivity of the OVH was higher than that of the LVH and CuVH as a result of the otolith-shaped microstructure. On the one hand, this design provides a high receiving area and moment of force when the sound wave is intercepted. On the other hand, hollow spheres can reduce the influence of shock.

**Table 2:** Comparison of the measured equivalent pressure sensitivity and calculated pressure sensitivity under a static state.

Frequency (Hz)	Receiving sensitivity *S*_*x*_ (dB)				20 log(*kd*) (dB)	Equivalent pressure sensitivity *S*_*p*_ (dB)			
	OVH	LVH	CuVH	WIVH		OVH	LVH	CuVH	WIVH
20	−214.4	−219.0	−220.6	−229.5	−41.5	−172.9	−177.5	−179.1	−188.0
31.5	−213.5	−213.5	−217.5	−224.0	−37.6	−175.9	−175.9	−179.9	−186.4
40	−211.7	−211.5	−216.0	−220.7	−35.5	−176.2	−176.0	−180.5	−185.2
50	−208.3	−209.7	−214.0	−219.2	−33.6	−174.7	−176.1	−180.4	−185.6
63	−207.0	−208.0	−212.5	−218.0	−31.6	−175.4	−176.4	−180.9	−186.4
80	−203.3	−205.7	−211.8	−216.3	−29.5	−173.8	−176.2	−182.3	−186.8
100	−202.1	−205.0	−209.2	−215.4	−27.6	−174.5	−177.4	−181.6	−187.8
125	−199.0	−203.6	−208.0	−214.1	−25.6	−173.4	−178.0	−182.4	−188.5
160	−194.7	−201.8	−206.0	−212.7	−23.5	−171.2	−178.3	−182.5	−189.2
200	−191.7	−199.2	−204.7	−211.4	−21.5	−170.2	−177.7	−183.2	−189.9
Average equivalent pressure sensitivity *S*_*p*_ (dB)						−173.8	−177.0	−181.3	−187.4
Difference in the average equivalent pressure sensitivity compared to that of the OVH (dB)						–	−3.2	−7.5	−13.6
Maximum stress on the beam via simulation (kPa)						131.7	114.8	58.1	35.2
Calculated pressure sensitivity under a static state *S*_*p_c*_ (dB)^a^						−157.0	−158.2	−164.1	−168.5
Difference in the calculated pressure sensitivity under a static state compared to that of the OVH (dB)						–	−1.2	−7.1	−11.5

^a^$S_{p\_c} = 20{\mathrm{log}}\left( {\frac{{\pi _l\sigma _{{\mathrm{max}}V_{{\mathrm{in}}}\,A}}}{{M_{{\mathrm{ref}}}}}} \right)$, *π*_*l*_ is the piezoresistance coefficient, *σ*_max_ is the maximum stress on the beam, *V*_in_ is the input voltage, *A* is the amplification factor, and *M*_ref_ is the reference sensitivity of 0 dB (1 V/μPa).

### Microfabrication process of the OVH

**Fig. 5:**
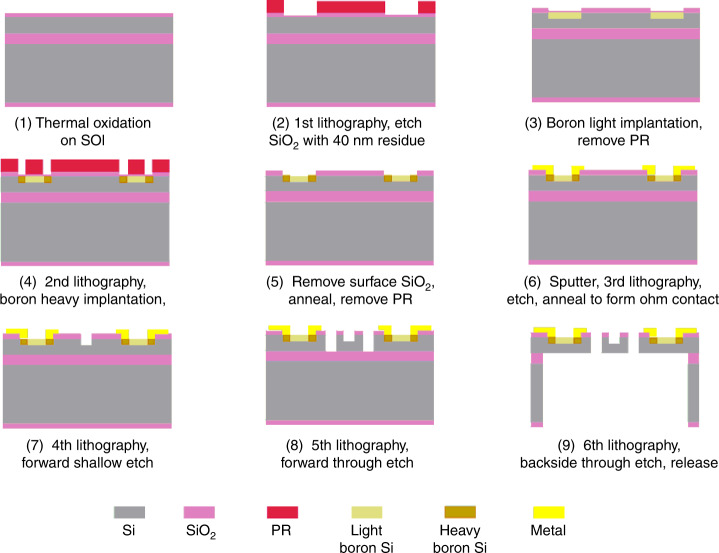
Sketch of the microfabrication process. (1) Thermal oxidation; (2) 1st lithography, Etch SiO_2_ with 40 nm residue; (3) Boron light implantation, remove photoresist; (4) 2nd lithography, Boron heavy implantation; (5) Remove surface SiO_2_, anneal, remove photoresist; (6) Sputter, 3rd lithography, etch the metal, anneal to form Ohm contact; (7) 4th lithography, forward shallow etch; (8) 5th lithography, forward through etch; (9) 6th lithography, backside through etch, release the structure.

The critical component of the MEMS hydrophone is the cross beam, where the piezoresistors are distributed. The dimensional parameters of the cross beam have a direct influence on the performance of the hydrophone. Hence, the cross beam is fabricated via the MEMS manufacturing process. The specific process is illustrated in Fig. 5.

### Calibration method

To verify the feasibility of the OVH, a sensitivity/directivity test is accomplished in a standing wave calibration system. The output voltage of the OVH is compared with that of a reference hydrophone to obtain the sensitivity of the OVH, which is calculated by:

8 $$M_x = \frac{{e_x}}{P}\frac{{\sin kd_r}}{{\cos kd_M}}$$

where *M*_*x*_ is the sensitivity, *e*_*x*_ is the output voltage of the hydrophone, *k* is the wavenumber (*k* = *ω*/*c*), *ω* is the circular frequency, *c* is the sound wave velocity in water, and *d*_*M*_ and *d*_*r*_ are the distances from the water surface to the OVH and the reference hydrophone, respectively. *P* can be obtained by measuring the output voltage of the reference hydrophone^17,18^.

Furthermore, the receiving sensitivity of MEMS hydrophone *S*_*x*_ is given by:

9 $$S_x = 20{\mathrm{log}}\left( {\frac{{e_x/p}}{{M_{{\mathrm{ref}}}}}{\mathrm{tan}}kd} \right)$$

Here, *M*_ref_ is the reference sensitivity as 0 dB (1 V/μPa), and *d* is the distance from the water surface to the vector hydrophone and reference hydrophone, both of which are set at the same distance.

In this experiment, *d* is set as 0.1 m. The frequency ranges from 20 to 200 Hz, corresponding to $kd \in \left[ {0.009,0.088} \right]$, where $\tan \left( {kd} \right) \approx kd$ according to Taylor’s formula:

10 $$S_x = 20\log \left( {\frac{{e_x/p}}{{M_{{\mathrm{ref}}}}}kd} \right) = 20\log \left( {\frac{{e_x/p}}{{M_{{\mathrm{ref}}}}}} \right) + 20{\mathrm{log}}\left( {kd} \right) = S_p + 20\log \left( {kd} \right)$$

*S*_*p*_ can be considered the equivalent pressure sensitivity of the MEMS hydrophone.

## Results

**Fig. 6:**
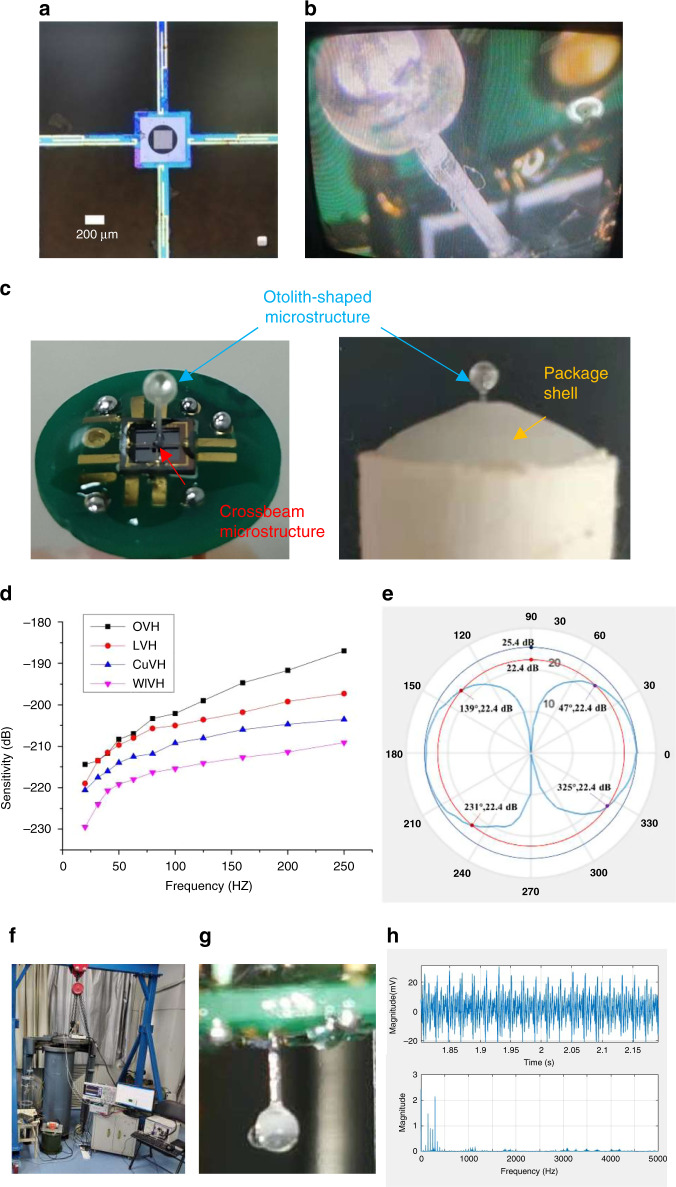
Measurement results of the OVH. **a** Microscopy photograph of the cross-beam microstructure. Cross beam, piezoresistors, metal lines, and shallow groove can be seen; **b** picture of an otolith-shaped cilium mounted on a beam; **c** picture of the chip on the PCB and in the shell; the otolith-shaped cilium has been mounted vertically on the center of the cross beam; **d** receiving sensitivity–frequency response curve; **e** directivity pattern at 100 Hz with 3 dB polar width of the OVH at 87°; **f** setup of the 10 MPa hydrostatic pressure measurement; **g** otolith-shaped microstructure after the 10 MPa test; **h** data acquisition under 10 MPa

A microscopy photograph of the microstructure is shown in Fig. 6a. The cross beam is formed in the suspending state. The piezoresistors are distributed on the beam. The metal lines are intact. The shallow groove at the center can be observed, which is favorable in cilia alignment and integration.

The otolith-shaped microstructure, with a hollow buoyant sphere on the top of the rod, was manufactured by microprecision 3D printing equipment based on projection microstereolithography technology (BMF microArch P130, up to 2 μm resolution). Otolith-shaped cilia with UV-curable glue were mounted on the central hole of the cross beam by a customized alignment setup, as shown in Fig. 6b.

A physical photograph of the OVH is shown in Fig. 6c. Three-micrometer-thick Parylene was deposited conformally on the cilium and beam by SCS PDS 2010 to ensure electrical insulation and structural protection. The otolith-shaped cilium was mounted vertically on the center of the cross beam. Finally, the chip with the processing circuit was packaged in a shell.

The receiving sensitivity–frequency response curves of various MEMS hydrophones are shown in Fig. 6d. The equivalent pressure sensitivity *S*_*p*_ over the frequency range of interest can be calculated thorough Formula (8), which is illustrated in Table 2. It can be seen that the average equivalent pressure sensitivity *S*_*p*_ over the frequency range of interest of the OVH reaches −173.8 dB (0 dB@1 V/μPa), an increase of 3.2 dB compared with that of the LVH, 7.5 dB compared with that of the CuVH, and 13.6 dB compared with that of the WIVH. The measurement results are in accordance with the simulation results extracted from Fig. 3b, as shown in Table 2. The absolute values of the calculated pressure sensitivity in the static state deviate from those of the measured average equivalent pressure sensitivity. This may be because calculation at the static state ignores the influence of dynamic damping. This deviation may also be attributed to the effect of package structure, noise of the reference hydrophone, and misestimation of the piezoresistance coefficient. Further investigation on the differences between the theoretical and experimental results should be performed. It should be noted that the differences in the calculated pressure sensitivity at the static state compared to the case of the OVH are consistent with those of the measured average equivalent pressure sensitivity.

The directivity pattern of the OVH at 100 Hz is shown in Fig. 6e, exhibiting typical cosine directivity. The 3 dB polar width of the OVH is measured as 87°, which shows superiority compared with 96° for the LVH, 89° for the CuVH, and 91° for the WIVH. This means that the OVH would perform better in distinguishing the sound along the sensitive axis than the LVH and slightly better than the CuVH and the WIVH.

Measurement under hydrostatic pressure was performed, as shown in Fig. 6f, including the equipment to implement 10 MPa hydrostatic pressure and data acquisition. The vibration motor was mounted to the hydrostatic pressure tube and utilized as a stimulating source. With 10 MPa hydrostatic pressure applied on the OVH, the otolith-shaped microstructure maintained its original shape without transformation, as shown in Fig. 6g. Data were acquired when the OVH was under the environment of 10 MPa hydrostatic pressure and vibration motor operation. As shown in Fig. 6h, the vibration signal could be distinguished in the time zone, and the peak appeared at 297.3 Hz in the frequency zone, which was in accord with the resonance frequency of the OVH. These results indicate that the OVH could work well under 10 MPa, owing to the open structure and Parylene encapsulation.

## Discussion and conclusions

In this paper, optimizations of cilium structure are made in order to realize high-sensitivity and low-frequency underwater acoustic detection, resulting in an otolith-shaped microstructure. Different parameters of the cilium structure influence the stress distribution and resonance frequency, which are analyzed via a theoretical model and simulation. Stress distributions of the different hydrophones are contrasted through simulations. Shock-resistance analysis shows that the OVH can resist higher shock than the LVH owing to otolith-shaped microstructure. Then, the fabrication process of the OVH is demonstrated. Finally, the OVH is tested in a standing wave field. The results show that the average equivalent pressure sensitivity *S*_*p*_ over the frequency range of interest of the OVH reaches −173.8 dB (0 dB@1 V/μPa), an increase of 3.2 dB compared with that of the LVH, 7.5 dB compared with that of the CuVH, and 13.6 dB compared with that of the WIVH. Additionally, the OVH has a cosine directional pattern with a 3 dB polar width of 87°.

Measurement under hydrostatic pressure indicates that OVH could be feasible under 10 MPa, owing to the open structure and Parylene encapsulation. The test results agree with the theoretical and simulation analysis, which verifies the feasibility and advancements of the OVH in detecting weak low-frequency underwater acoustic signals.

## Supplementary information

Supplemental Material
