# Supplementary material for: Design and implementation of a jellyfish otolith-inspired MEMS vector hydrophone for low-frequency detection
Source: Microsyst Nanoeng. 2021 Jan 1;7:1. doi: 10.1038/s41378-020-00227-w (PMC8433173; doi:10.1038/s41378-020-00227-w)
Supplement: Supplementary file 1 — Supplemental Material [file 41378_2020_227_MOESM1_ESM.docx]

**Design and implementation of** **a jellyfish otolith-inspired MEMS vector hydrophone for low-frequency detection**

Renxin Wang^1^, Wei Shen^1^, Wenjun Zhang^1^, Jinlong Song^1^, Nansong Li^2^, Mengran Liu^3^, Guojun Zhang^1^, Chenyang Xue^1^, Wendong Zhang[[1]](#footnote-1),*

1 State Key Laboratory of Dynamic Testing Technology, North University of China, Taiyuan, China

2 College of underwater acoustic engineering, Harbin Engineering University, Harbin, China

3 Hubei Key Laboratory of Modern Manufacturing Quantity Engineering, School of Mechanical Engineering, Hubei University of Technology, Wuhan, Hubei, China

* Correspondence: Prof. Wendong Zhang, Email: wdzhang@nuc.edu.cn

Analysis on resonance frequency and working band

As for MEMS hydrophone, -2/3 octave of resonance frequency in the water was usually used as the upper limit of bandwidth. Resonance frequency of microstructure is simulated as 527Hz in the air and 314 Hz in the water, and upper limit of working band is estimated as about 200Hz. Specific analysis is demonstrated as following.

When the cilium vibrates in the fluid, it suffers from the resistance of fluid $F_{\mathrm{fluid}}$, which consists of two parts: one part is vibration damping force of fluid, which is correlated positively to fluid viscosity; the second part is the force that drives the vibration of surrounding fluid mass, which is dependent on fluid viscosity. $F_{\mathrm{fluid}}=-m_{\mathrm{fluid}}\ddot{x}{-k}_{\mathrm{fluid}}\dot{x}$, where *x* is the deflection of the cilium, $k_{fluid}$ is the fluid damping coefficient, and $m_{\mathrm{fluid}}$ is the added fluid mass. $k_{\mathrm{fluid}}$ and $m_{\mathrm{fluid}}$ are related to the density, viscosity, structure size.

Adding mass of otolith-shaped cilium could be figured out as $m_{\mathrm{fluid}}=\rho_{f}(4.187R^{3}+3.14d^{2}h)$ , where $\rho_{f}$ is density of fluid, *R* is outer radius of sphere, *d* is radius of rod and *h* is height of rod. And mass of otolith-shaped cilium could be figured out as $m=\rho_{c}(\frac{4}{3}\pi(R^{3}-r^{3})+\pi d^{2}h)$, where *r* is inner radius of sphere.

For the second-order oscillatory system, the motion can be described as the differential equation of motion mass - spring vibration model with damped force.

When the cilium vibrates driving by external simple harmonic force $P_{0}\sin\omega t\text{ }$, the kinematic equation could be deduced:

$\ddot{x}+2\zeta\omega_{0n}\dot{x}+\omega_{0n}^{2}x=\frac{P_{0}}{m+m_{\mathrm{fluid}}}\sin$($\omega t$) (s1)

where *ζ* is damping ratio, $\zeta=\frac{k_{\mathrm{fluid}}}{2(m+m_{\mathrm{fluid}})\omega_{0n}}$ ，$\omega_{0n}^{2}=\frac{m\omega_{0}^{2}}{m+m_{\mathrm{fluid}}}$, $\omega_{0}$is the natural frequency without damping, *m* is the mass of cilium.

Therefore, the amplitude of frequency response can be obtained.

$\mathrm{M}(\omega)=[(1-\frac{\omega^{2}}{\omega_{0n}^{2}})^{2}+4\zeta^{2}\frac{\omega^{2}}{\omega_{0n}^{2}}]^{-\frac{1}{2}}\text{ }$ (s2)

Resonance frequency is related to the damping.

$\omega_{r}=\sqrt{1-2\zeta^{2}}\omega_{0n}={\sqrt{\frac{m(1-2\zeta^{2})}{m+m_{\mathrm{fluid}}}}\omega}_{0}=0.68\sqrt{1-2\zeta^{2}}\omega_{0}$ $0\leq\zeta\leq0.707$ (s3)

Normally, the amplitude of frequency response $20\mathrm{logM}(\omega)\leq3\text{dB}$ is considered as the available area that isn’t influenced by resonance frequency.

Therefore, it can be obtained:

$\frac{\omega^{2}}{\omega_{r}^{2}}\leq[1-\sqrt{1-\frac{0.5}{(1-2\zeta^{2})^{2}}}]\text{ }$ (s4)

$\zeta$could be measured by damped free vibration experiments, $\zeta=\frac{\omega_{f}}{2\pi n\omega_{0n}}\text{In}\frac{x_{k}}{x_{k+n}}$, $x_{k}$ is the amplitude of the *k*th vibration cycle, which accords with exponential decay function $\text{A}e^{-\zeta\omega_{0n}t}$. Here, $\zeta$is estimated about 0.2.


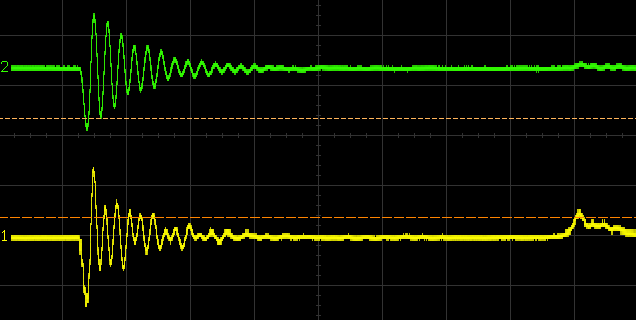


Fig.S1 Damped free vibration experiment

Finally, we could obtain $\omega_{r}=0.63\omega_{0}$. Considering that resonance frequency $\omega_{0}$ in the air is simulated as 527Hz, $\omega_{r}$would be 332Hz, corresponding the simulated result 314Hz in the water. On the other hand, $\frac{\omega}{\omega_{r}}\leq0.6$, corresponding to abovementioned -2/3 octave (about 0.63). The upper limit of working band could be determined as about 200Hz, 0.6 time of resonance frequency in the water.

1. 1 State Key Laboratory of Dynamic Testing Technology, North University of China, Taiyuan, China

   2 College of underwater acoustic engineering, Harbin Engineering University, Harbin, China

   3 Hubei Key Laboratory of Modern Manufacturing Quantity Engineering, School of Mechanical Engineering, Hubei University of Technology, Wuhan, Hubei, China

   * Correspondence: Prof. Wendong Zhang, Email: wdzhang@nuc.edu.cn [↑](#footnote-ref-1)
